# Effects of estradiol and 11-ketotestosterone pre-treatment on artificial induction of maturation in silver female shortfinned eels (*Anguilla australis*)

**DOI:** 10.1371/journal.pone.0229391

**Published:** 2020-02-24

**Authors:** Erin L. Damsteegt, Georgia Thomson-Laing, Matthew J. Wylie, P. Mark Lokman

**Affiliations:** Department of Zoology, University of Otago, Dunedin, New Zealand; Shanghai Ocean University, CHINA

## Abstract

Our previous work documented significant advancements in steroid-induced progression of oogenesis, demonstrating that co-treatment of female eels with 11-ketotestosterone (11KT) and estradiol-17β (E_2_) successfully induced uptake of vitellogenin by oocytes. Here we evaluate the effects of this steroid co-treatment on subsequent time to ovulation and egg quality in shortfinned eels artificially matured by hypophysation. Co-treatment with 11KT (1 mg) and E_2_ (0.2 or 2 mg) significantly reduced time to ovulation and therefore, the amount of pituitary homogenate required, without any detrimental effects on gonadosomatic index, oocyte diameter or the total weight of stripped eggs. E_2_ treatment resulted in promising increases in fertilization rates. These indicators suggest that co-treatment with 11KT and E_2_ holds promise for future artificial maturation practices in terms of minimising fish handling and stress, and of reducing the need for expensive pituitary preparations.

## Introduction

The reliable production of good quality eggs is a well-known bottleneck plaguing much of the aquaculture industry [1–3]. Indeed, generating high quality eggs from captivity-reared eels is an increasingly urgent necessity for the development of eel propagation practices world-wide [4,5]. The well-documented gonadal arrest of captive eels demands that fish must be frequently manipulated with fertility drugs in order to advance oocytes past the early vitellogenic stage. Traditionally, this has been achieved by hypophysation, i.e., injections of either carp or salmon pituitary homogenates (CPH or SPH, respectively) [6–14]. This approach has allowed for successful acquisition of larvae from captive eel stocks but the disadvantages are several-fold: pituitary homogenates are crude, very expensive and eels require weekly injections to progress oocytes to the pre-ovulatory stage. Weekly handling is not only costly in terms of human resources but being removed from the tank and injected under anaesthesia is inherently stressful for the eels. Furthermore, this method is also typically associated with reduced egg quality and low larval survival [5]. A variety of approaches has been explored to improve hypophysation protocols including simulated migration [15], feminising broodstock [16–18] or using androgen pre-treatments [19,20].

Recent efforts using both aromatizable and non-aromatizable androgens (17-methyltestosterone, 17MT; androstenedione, AD4; 11-ketotestosterone, 11KT) as pre-treatments to hypophysation-induced artificial maturation have been promising. Following seven weeks of pre-treatment with either 3 or 30 mg 17MT, shortfinned eels (*Anguilla australis*) took, on average, 45 days of CPH treatment to reach the pre-ovulatory stage [19], 30 days fewer than eels that received only CPH. Whilst 17MT pre-treatment thus showed promise for use in artificial maturation protocols, unusual cytology was observed in oocytes from treated fish. In the same study, no significant difference in the time required to reach the pre-ovulatory stage was found between eels pre-treated with either 1 mg AD4, 1 mg 11KT or a placebo, although a hastening trend was observed following 11KT treatment. A similar effect was seen in the European eel (*Anguilla anguilla*) in response to 1 mg implants of 17MT [20]. Furthermore, these researchers reported that treatment with 1 mg 17MT resulted in eels spawning significantly more eggs which had much higher fertilization (~90% vs ~71%) and hatch rates (~70% vs ~21%) than observed in eggs from control fish.

The encouraging results from the androgen pre-treatment studies, coupled with the known ability of estradiol-17β (E_2_) to induce hepatic vitellogenin production in teleosts [21–25], fuelled our interest in a combined androgen/estrogen pre-treatment regime. Our recently published work [26] clearly demonstrates the success of the combined pre-treatment in increasing ovarian yolk deposition and progressing oocytes from the early to mid vitellogenic stage in the absence of exogenous gonadotropin. In this follow-up study on the same fish, we evaluate the efficacy of E_2_ and/or 11KT as pre-treatment on hypophysation-induced artificial maturation in female *A*. *australis*.

## Methods

### 11KT and E_2_ pre-treatment

Fish handling was approved by the University of Otago Animal Ethics Committee and conducted in accordance with the guidelines of the Australian & New Zealand Council for the Care of Animals in Research and Teaching. All details of fish collection, husbandry and steroid pre-treatment are described in Thomson-Laing et al. [26]. Briefly, 36 early pubertal (‘silver’), wild-caught, female shortfinned eels (body weight: 748.7 ± 11.6 g; total length: 74.1 ± 0.6 cm; condition factor: 2.73 ± 0.05) were housed in 200L recirculating tanks at 10–15 ppt and 14–17 ^o^C on a 12:12 light regime. After acclimation, all eels were anesthetized in Aqui-S (0.3 mL/L: Aqui-S New Zealand Ltd) and implanted (abdominal cavity) with two slow-release, cholesterol-cellulose pellets (see: [19,27]), the first pellet containing either 0 or 1 mg of 11KT (Steraloids) and the second pellet containing either 0, 0.2 or 2 mg of E_2_ (Steraloids). Six eels were assigned to each treatment and treatment groups housed in separate tanks. At 12 weeks, all eels were ovary-biopsied (see: [26]), transferred to seawater and recruited into the induced spawning experiment described below.

### Artificial maturation of females

Each week, eels were immobilised in Aqui-S, weighed and injected intramuscularly with 10 mg/kg SPH. Treatment was repeated until eels had sufficiently increased in body weight (> 10%) and looked gravid, at which time ovarian tissue was collected by a 14G hypodermic needle inserted through the body wall (c.f., [28]). Using observations by Unuma et al. [29] as a guide, if Stage 4 clearing oocytes were observed then eels received either 2000 IU/kg human chorionic gonadotropin (hCG; MSD Animal Health) or 40 mg/kg SPH to mimic the pre-ovulatory luteinizing hormone surge. Fish were then placed into a separate tank held at 20 ^o^C for monitoring. After 12–24 h, eels were biopsied again and if oocytes had reached stage 6/7 [29], they received a single dose of 2 mg/kg 17, 20β-dihydroxy-4-pregnen-3-one (DHP; Steraloids) administered intraperitoneally. To ensure that DHP was not localized to a single injection site, the total volume was administered across 4 different sites, from just posterior from the liver, to peri-anally.

### Sample collection

Eggs were hand-stripped (S1 Video) and weighed before the eel was euthanised (0.3 g/L benzocaine; Sigma-Aldrich). Following dissection, remaining ovarian tissue was removed and weighed, and the combined weight of the stripped eggs and ovarian tissue used to calculate the gonadosomatic index (GSI) (organ weight/total body weight). Liver weight was also recorded for calculation of the hepatosomatic index (HSI).

### Artificial maturation of males

Eighteen male eels (body weight: 148 ± 16 g) were housed in a single 200L recirculating tank in full-strength seawater at 20 ^o^C on a 12:12 light regime. Maturation was induced by hCG injection as described in Lokman and Young [7].

### Egg buoyancy and fertilization rates

Sperm was collected in plastic syringes by gently squeezing the dried abdomen of matured males. Upon egg collection, sperm were activated in seawater and promptly added to the eggs. Eggs and sperm were mixed by hand and left to stand for 5 min. After transfer onto a nylon sieve submerged in seawater, eggs were washed with fresh seawater. They were then transferred to a 1 L measuring cylinder to allow the proportion of buoyant eggs (float rate), to be estimated.

A sample of buoyant eggs was incubated in 500 mL plastic containers maintained at 19–22 ^o^C and a subsample of 50–150 eggs was used to estimate fertilization rate at the 2- to 4-cell stage. In cases where all eggs sunk following transfer to a 1 L measuring cylinder, eggs were still incubated and fertilization rates estimated as above.

Attempts were made to fertilize a portion of stripped eggs from all fish. However, as females matured quicker than anticipated and males initially produced immotile sperm, useable data could be only gained from fish that matured after eight weeks of SPH treatment i.e. those not implanted with 11KT.

### Statistical analysis

Data are presented as means and standard errors. Differences between treatment groups were identified using two-way ANOVA (dose of E_2_ (3 levels) and dose of 11KT (2 levels)) as factors in Graphpad Prism8. Prior to analysis, all data were checked for homoscedasticity and normality using Levene’s and Shapiro-Wilk tests, respectively. Data that violated either assumption (time to pre-ovulatory stage, SPH dose required to pre-ovulatory stage and HSI) were log-transformed and the assumptions re-checked prior to ANOVA.

## Results

### Fish health

Three fish died during steroid pre-treatment (see: [26]). Only 2 of the remaining 33 eels did not reach the pre-spawning condition; one fish treated with 11KT + 0.2 mg E_2_ died following hCG injection, possibly due to a blocked gonopore, and another one (2 mg E_2_) did not respond to SPH injection and was euthanised after all other fish had spawned.

### Body weight changes following steroid implantation

**Fig 1:**
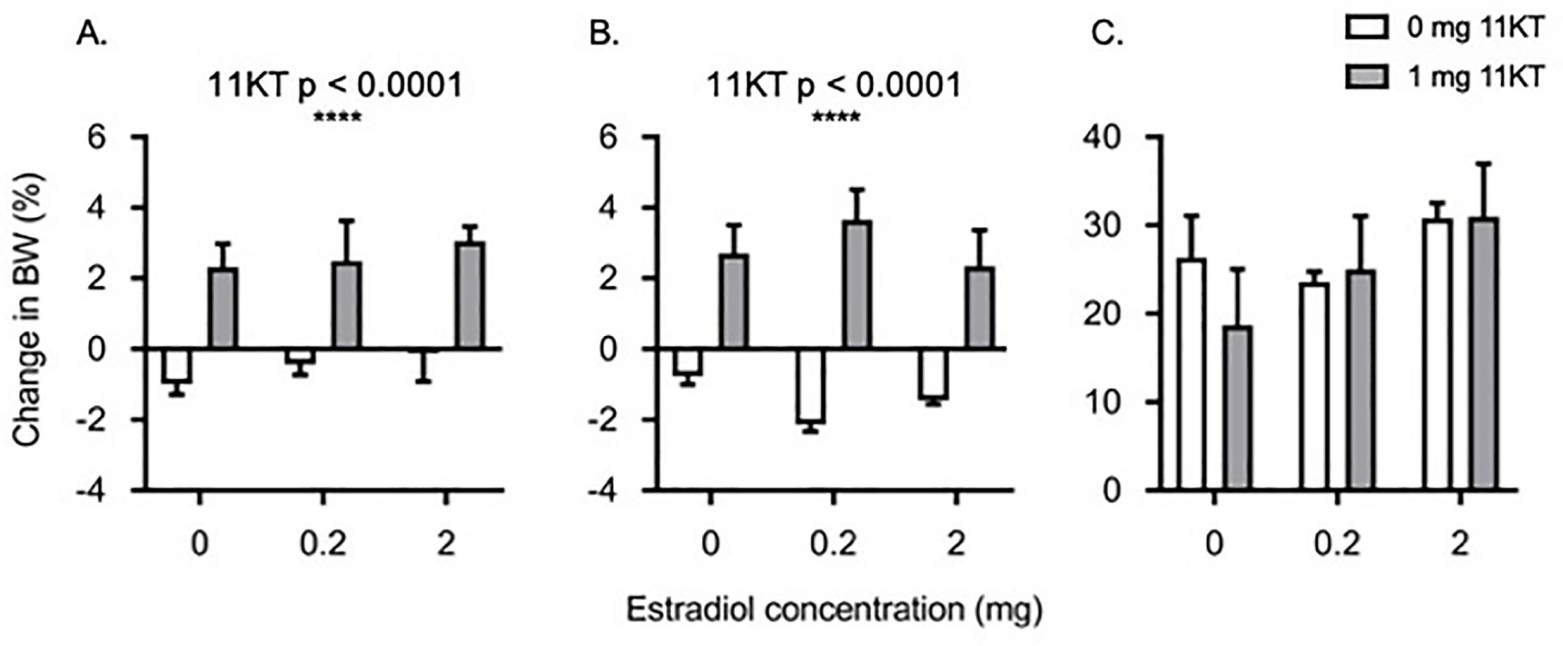
Changes in body weight following steroid pre-treatment. Effects of implantation with increasing doses of estradiol-17β (0, 0.2 and 2 mg) in combination with either 0 mg 11-ketotestosterone (11KT) (white bars) or 1 mg 11KT (grey bars) on the change in body weight (BW) of female shortfinned eels (*Anguilla australis*) after six (A) or twelve (B) weeks of steroid pre-treatment or at euthanasia (C). Significant differences are indicated on the figure. Bars represent means ± one standard error. For all treatment groups, n = 6 eels, except for the group treated with 2 mg E_2_ at 12 weeks (n = 4) and that treated with 0.2 mg E_2_ + 11KT at 12 weeks (n = 5).

Six weeks into the steroid pre-treatment, the majority of the eels that did not receive 11KT had lost weight (Fig 1A). In contrast, only a single 11KT-implanted fish had lost weight and all other 11KT-treated eels presented with an increase in body weight. These differences were even more pronounced after 12 weeks at which time all eels that were not implanted with 11KT had lost weight and all eels that were treated with 11KT had gained weight (Fig 1B). The weight loss in the fish that did not receive 11KT was completely recovered following SPH injection such that there was no difference in weights between eels in any of the treatment groups at the time the eggs were hand-stripped i.e. at euthanasia (Fig 1C).

### Total SPH dose required to reach pre-ovulatory stage

**Fig 2:**
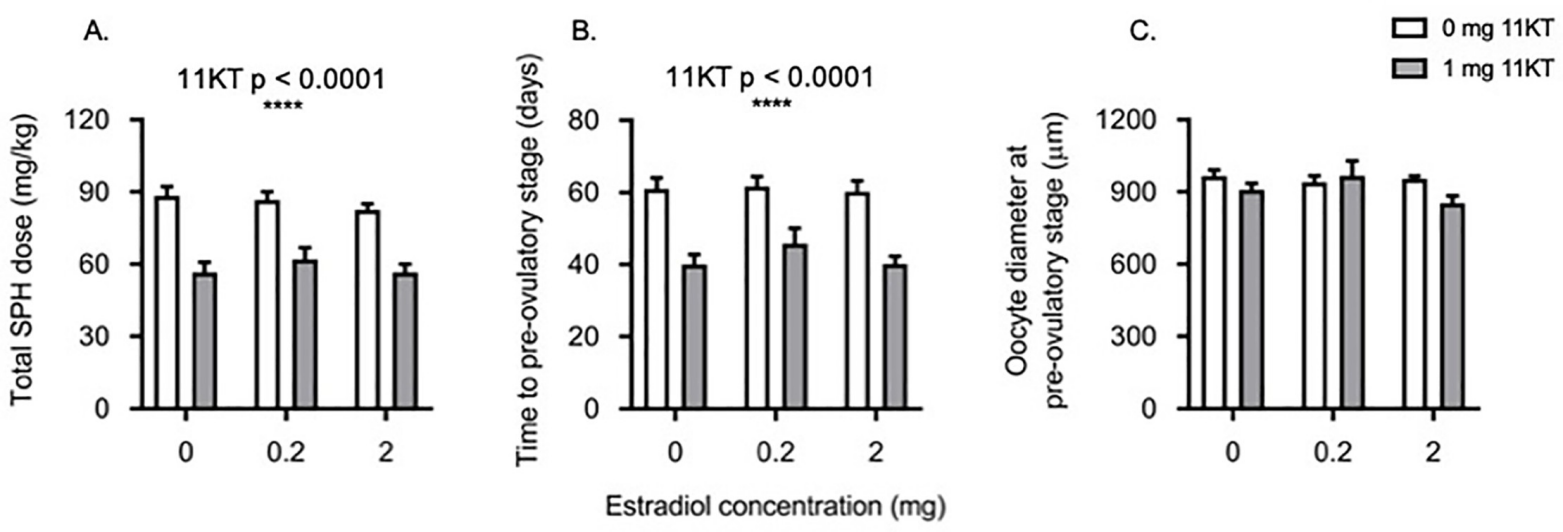
SPH dose, time and oocyte diameter at pre-ovulatory stage. Effects of implantation with increasing doses of estradiol-17β (0, 0.2 and 2 mg) in combination with either 0 mg 11-ketotestosterone (11KT) (white bars) or 1 mg 11KT (grey bars) on the total salmon pituitary homogenate (SPH) dose (A) and time (B) required for shortfinned eels (*Anguilla australis*) to reach the pre-ovulatory stage (i.e. injection with 17,20β-dihydroxy-4-pregnen-3-one (DHP)) and the diameter of the oocytes at the time of DHP injection (C). Significant differences are indicated on the figure. Bars represent means ± one standard error. For all treatment groups, n = 6 eels, except for the group treated with 2 mg E_2_ for all endpoints (n = 4), 0.2 mg E_2_ + 11KT for time and oocyte diameter at pre-ovulatory stage (n = 4) and SPH dose (n = 5) and steroid-free fish for time and oocyte diameter (n = 5).

11KT significantly reduced (F_1,27_ = 72.93; p < 0.0001) the amount of SPH required for fish to reach the pre-ovulatory stage (Fig 2A). One 11KT-only treated fish required just 40 mg of SPH until reaching this stage. A further five 11KT-treated (co-implanted with various doses of E_2_) fish reached the pre-ovulatory stage after having received only 50 mg of SPH. With the exception of a single fish which required 80 mg of SPH, all 10 remaining 11KT-treated (various doses of E_2_) individuals had transparent oocytes after either 60 or 70 mg of SPH. In contrast, eels that did not receive 11KT implants required between 80 and 100 mg of SPH to reach the pre-ovulatory stage. Treatment with E_2_ had no effect on the amount of SPH required to reach the pre-ovulatory stage.

### Time to reach pre-ovulatory stage

Treatment with 11KT significantly decreased (F_1,25_ = 57.03; p < 0.0001) the time taken for eels to reach the pre-ovulatory stage (Fig 2B). On average, eels treated with 11KT reached the pre-ovulatory stage 20 days sooner than their non-11KT treated counterparts, regardless of E_2_ dose.

### Oocyte morphometrics

**Fig 3:**
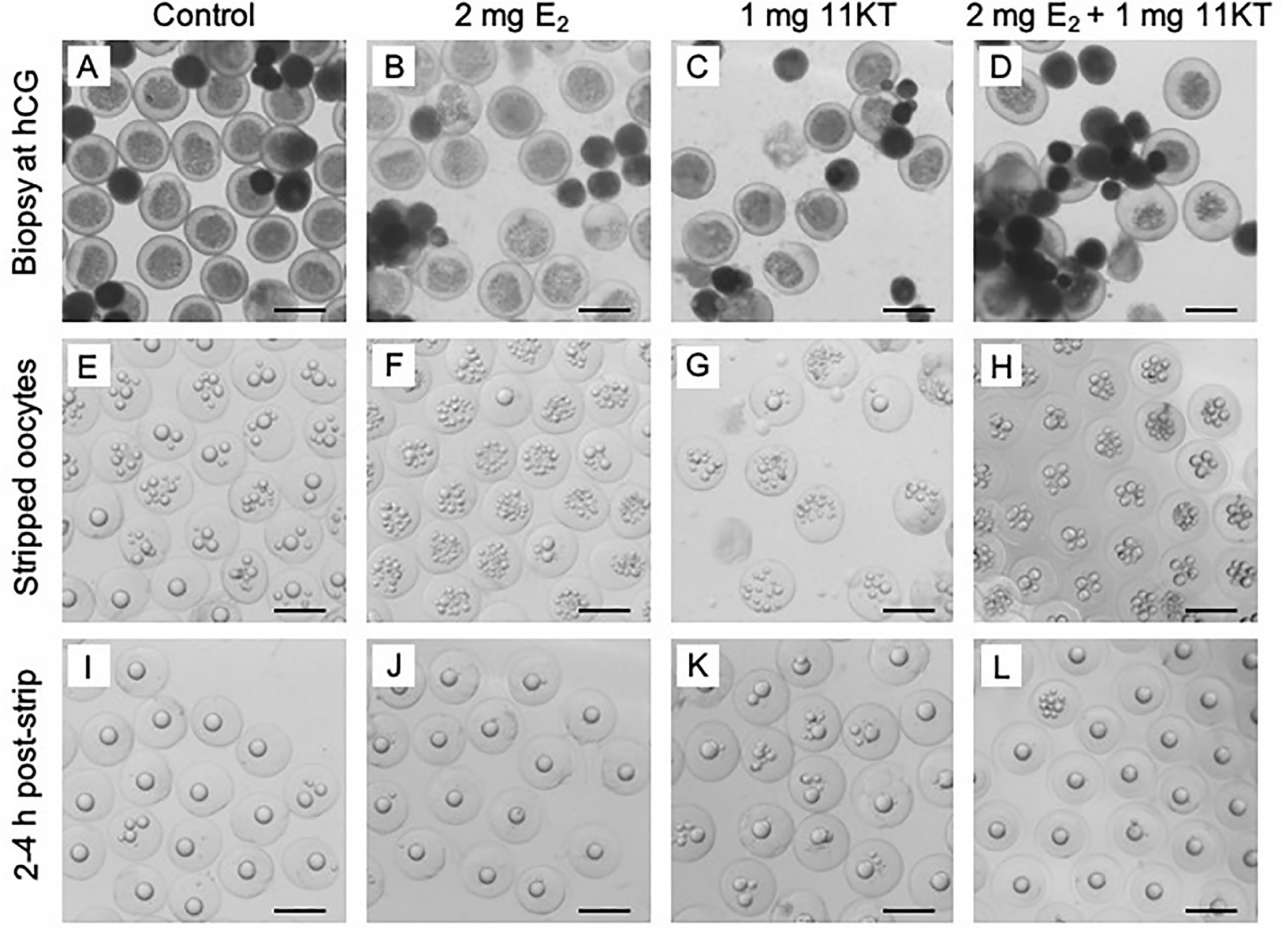
Micrographs of oocytes at biopsy, strip and 2–4 h post-strip. Ovarian biopsy at time of human chorionic gonadotropin (hCG) injection (A-D), eggs at strip-spawning (E-H) and eggs with coalesced oil droplets 2–4 h post-stripping (I-L) from shortfinned eels (*Anguilla australis*) treated with no steroid (A, E & I), 2 mg of estradiol-17β (B, F & J), 1 mg 11-ketotestosterone (C, G & K) or a combination of 2 mg of estradiol-17β and 1 mg 11-ketotestosterone (D, H & L). Scale bar = 1 mm. Each column of images was collected from germ cells/gametes from the same female.

Treatment with either steroid alone or in combination did not have any effect on the oocyte diameter at the pre-ovulatory stage (F_2,25_ = 1.95; p = 0.1637; Fig 2C). Oocyte diameters in steroid-free fish averaged 882 ± 30 μm (mean ± standard error) compared to 850 ± 20 μm in E_2_ treated fish, 822 ± 52 μm in 11KT treated fish and 807 ± 34 μm in fish that received the combined steroid treatment. Biopsied oocytes and ovulated eggs appeared morphologically similar between fish regardless of treatment (Fig 3).

### Spawned egg weights and somatic indices

**Fig 4:**
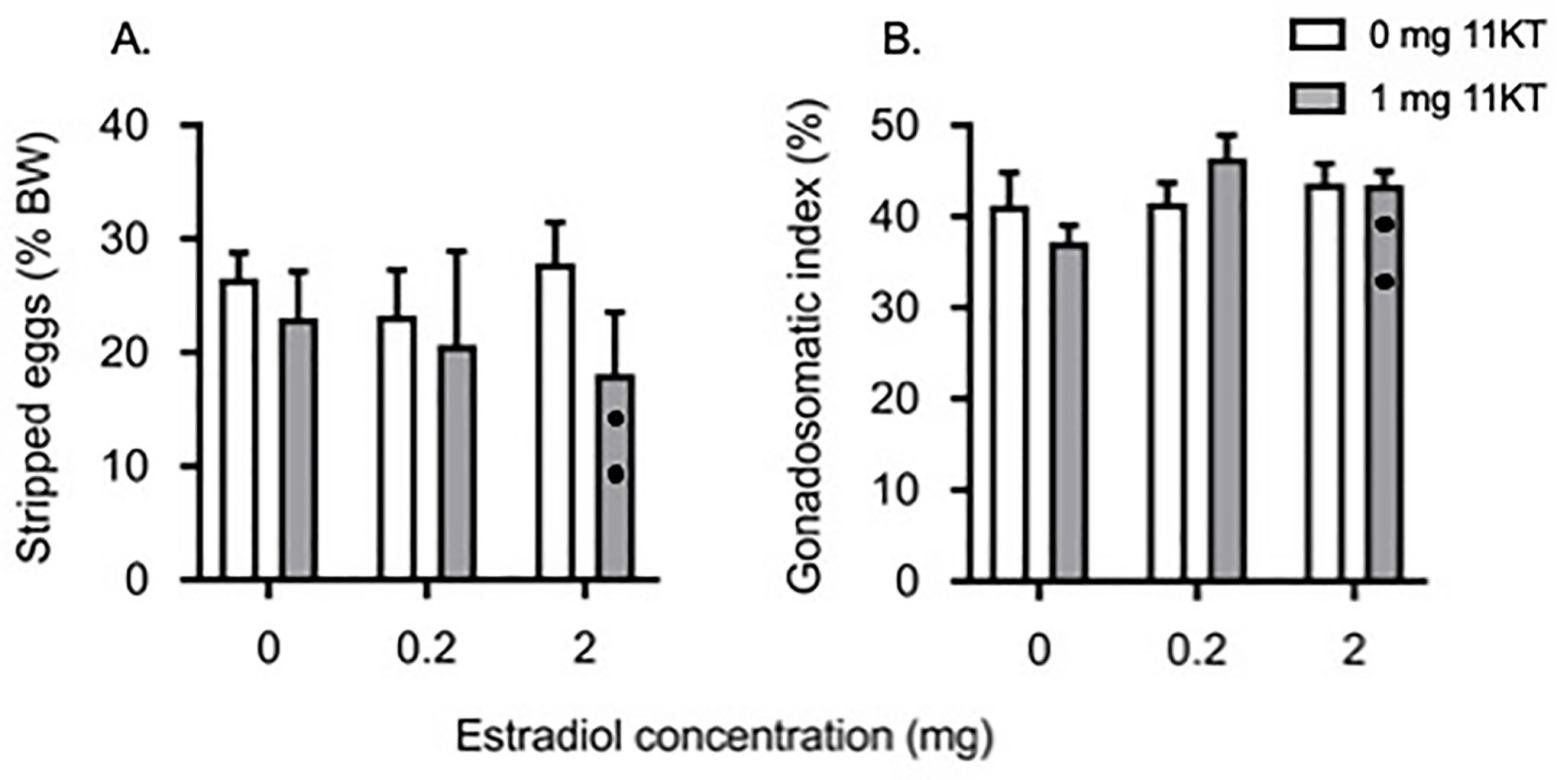
Weight of stripped eggs and gonadosomatic index. Effects of implantation with increasing doses of estradiol-17β (0, 0.2 and 2 mg) in combination with either 0 mg 11-ketotestosterone (11KT) (white bars) or 1 mg 11KT (grey bars) on the weight of stripped eggs as a percentage of body weight (A) and on the gonadosomatic index (stripped eggs and remaining ovarian tissue) (B) in shortfinned eels (*Anguilla australis*) artificially induced to mature. • indicate data points that were removed from statistical analysis after fish spontaneously spawned in tanks. Bars represent means ± one standard error. For all treatment groups, n = 6 eels, except for the group treated with 2 mg E_2_ for both endpoints (n = 4), 0.2 mg E_2_ + 11KT for weight of stripped eggs (n = 4) and GSI (n = 5), 1 mg 11KT for both endpoints (n = 5) and steroid-free fish for weight of stripped eggs (n = 5).

Pre-treatment with E_2_ alone or in combination with 11KT did not have any effect on the weight of the eggs stripped from the fish following ovulation (Fig 4A) or the GSI (Fig 4B). However, two fish from the 2 mg E_2_ + 11KT spontaneously spawned a large number of eggs into the tank before we were able to collect them, both fish were excluded from the analysis but are indicated on the figure.

**Fig 5:**
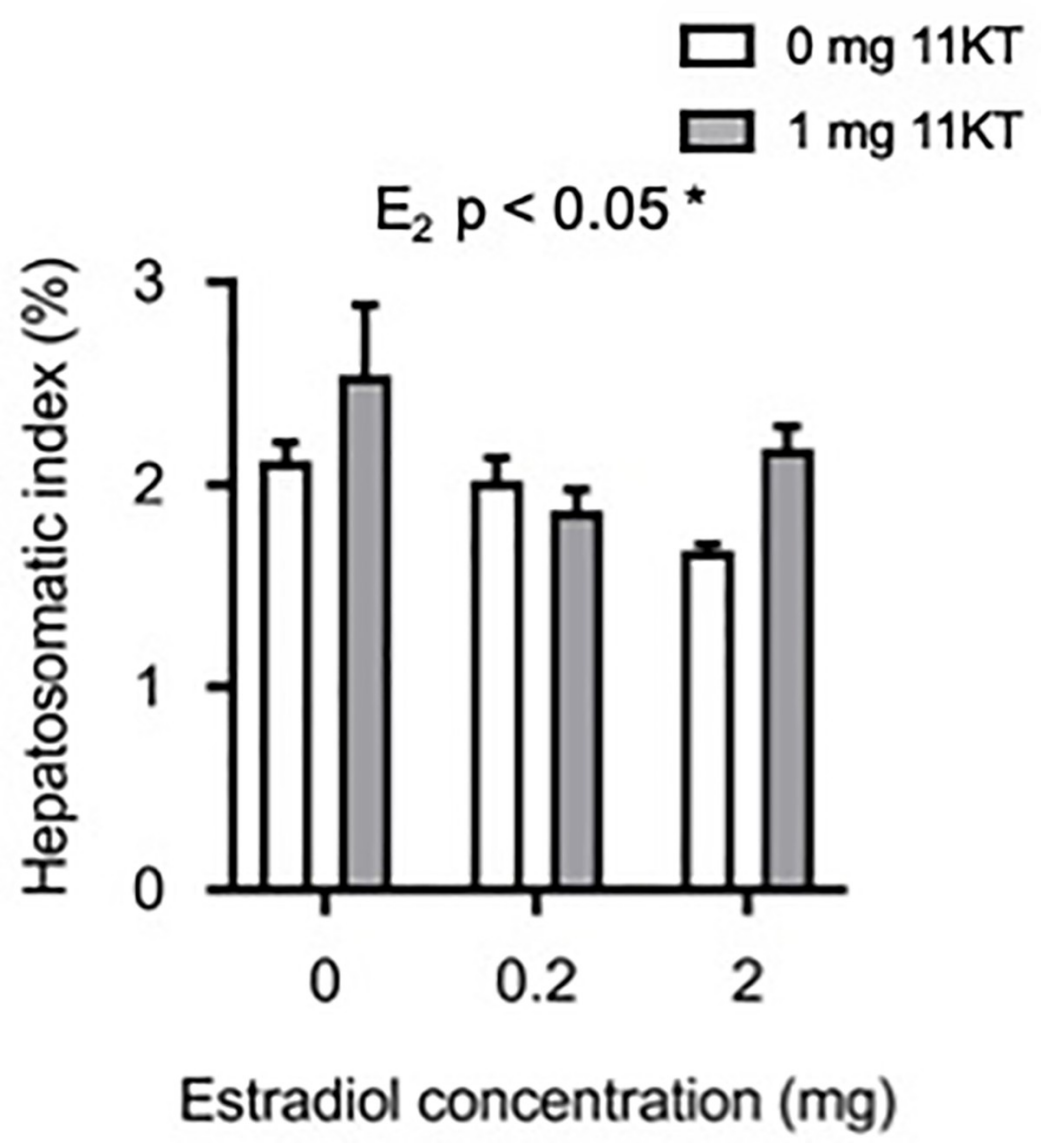
Hepatosomatic index. Effects of implantation with increasing doses of estradiol-17β (0, 0.2 and 2 mg) in combination with either 0 mg 11-ketotestosterone (11KT) (white bars) or 1 mg 11KT (grey bars) on hepatosomatic index of artificially matured eels (*Anguilla australis*) at euthanasia. Significant differences are indicated on the figure. Bars represent means ± one standard error. For all treatment groups, n = 6 eels, except for the group treated with 2 mg E_2_ (n = 4) and that treated with 0.2 mg E_2_ + 11KT (n = 5).

Treatment with E_2_ resulted in a significantly lower HSI (F_2,26_ = 4.31; p < 0.05) compared to eels that did not receive E_2_, regardless of whether they were also treated with 11KT (Fig 5). Eels that did not receive E_2_ had an average HSI of 2.31 ± 0.16% compared to eels that received either 0.2 mg or 2 mg E_2_ which had HSIs of 1.95 ± 0.08% and 1.98 ± 0.10%, respectively.

### Egg buoyancy and fertilization rates

Float rate estimates were not statistically analysed due to issues with clean seawater availability. When forced to use low quality seawater at the time of fertilization, eggs did not hydrate well, the perivitelline space was narrow and the eggs sunk. These issues did not appear detrimental to fertilization rates as 100% of the eggs from the fish with the highest fertilization rate (79%) sunk.

**Fig 6:**
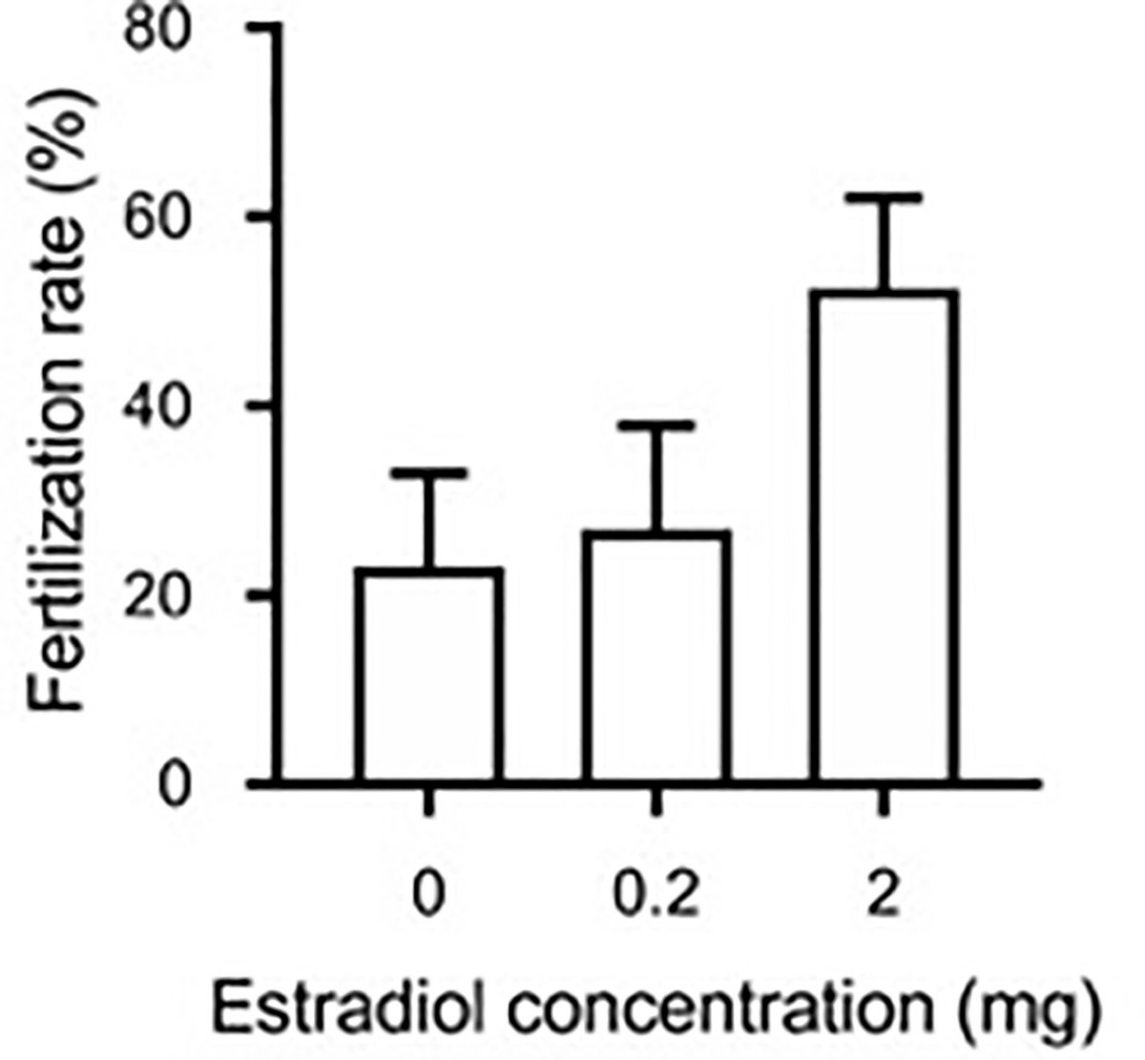
Fertilization rate. Fertilization rates of eggs stripped from shortfinned eels (*Anguilla australis*) that were implanted with increasing doses of estradiol-17β (0, 0.2 and 2 mg). Bars represent means ± one standard error. For all treatment groups, n = 6 eels, except for the group treated with 2 mg E_2_ (n = 4) and steroid-free fish (n = 5).

Fertilization rates showed an increasing trend (F_2,12_ = 2.03; p = 0.17) as the dose of E_2_ increased (Fig 6). On average, fish that did not receive any pre-treatment had fertilization rates of 23 ± 10%. Fish that received the low or high dose of E_2_ had average fertilization rates of 27 ± 11% and 52 ± 9% respectively. Four E_2_ treated fish had fertilization rates above 50% with one reaching 79%. Eggs were obtained from 15 females (all 0 mg 11KT) at the time that motile sperm was also available and only three (two 0 mg E_2_ treated fish and one 0.2 mg E_2_ treated fish) of those females produced eggs with fertilization rates below 5%.

### Hatch rates and larval survival

Due to the lack of larval rearing facilities we did not attempt to quantify hatch rates or larval survival in this study. However, hatching was observed for at least some eggs from nearly all batches; in a few instances, eggs hatched *en masse* (estimated at ~80% of the proportion of fertilized eggs). Gross morphology of the larvae appeared normal regardless of maternal treatment group; however, no larvae were obtained from the 11KT-only group due to the lack of good quality sperm at the time of egg retrieval.

### Anecdotal observations

Whilst the employed time between hCG and DHP injections was very consistent (24.2 ± 0.2 h), the time between DHP injection and egg collection (either spontaneous spawning or hand-stripping) varied between fish that spawned spontaneously and/or stripped well (15.6 ± 0.4 h) and fish that stripped poorly or not at all (19.9 ± 0.7 h). The shortest latency time to egg collection was 13 h. We were not able to collect eggs from any fish that had not responded to DHP injection after 19 h.

At the time of biopsy, the oocytes from four fish had progressed too far to administer hCG, and these fish were instead injected with DHP. All four fish subsequently hand-stripped well; two were hand-stripped at a time when we did not have good quality sperm available, eggs from the third fish were not successfully fertilized, and eggs from the fourth fish had a fertilization rate of 36%.

**Fig 7:**
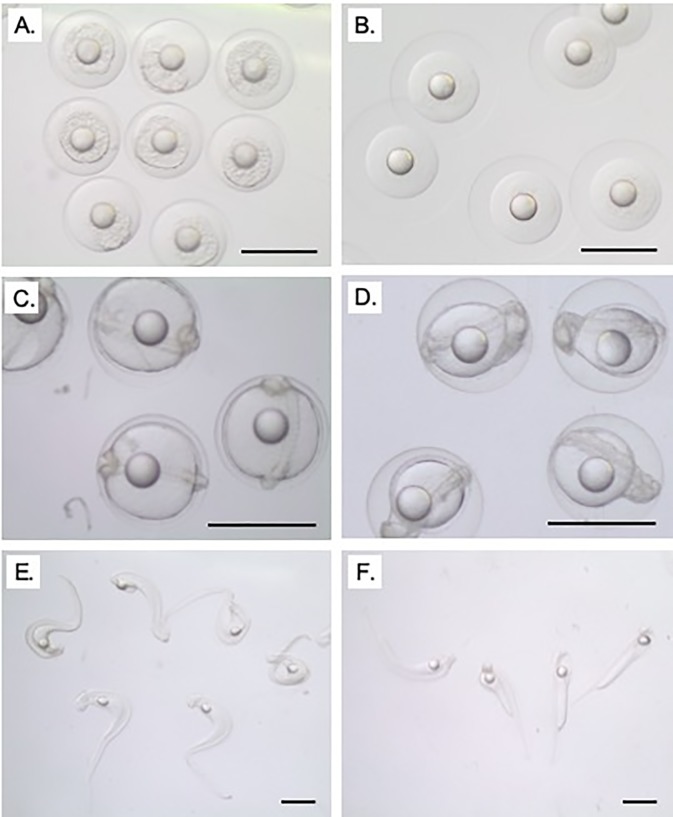
Larval development. Micrographs of *Anguilla australis* larval development in poorly hydrated eggs that sunk (A, C and E) and in well-hydrated, buoyant eggs (B, D and F). Larvae mechanically-hatched from poorly hydrated eggs (E) and larvae that hatched unaided from well hydrated eggs (F). Scale bar = 1 mm.

The aforementioned issues with the quality of the seawater not only dictated the eggs’ ability to hydrate and therefore, gain buoyancy, but the flow-on effects appeared to influence the larvae’s ability to hatch unaided. Larvae from poorly hydrated eggs could be hatched mechanically (forceps) or enzymatically (chorionase). However, those that survived assisted hatching were often kinked (Fig 7).

**Fig 8:**
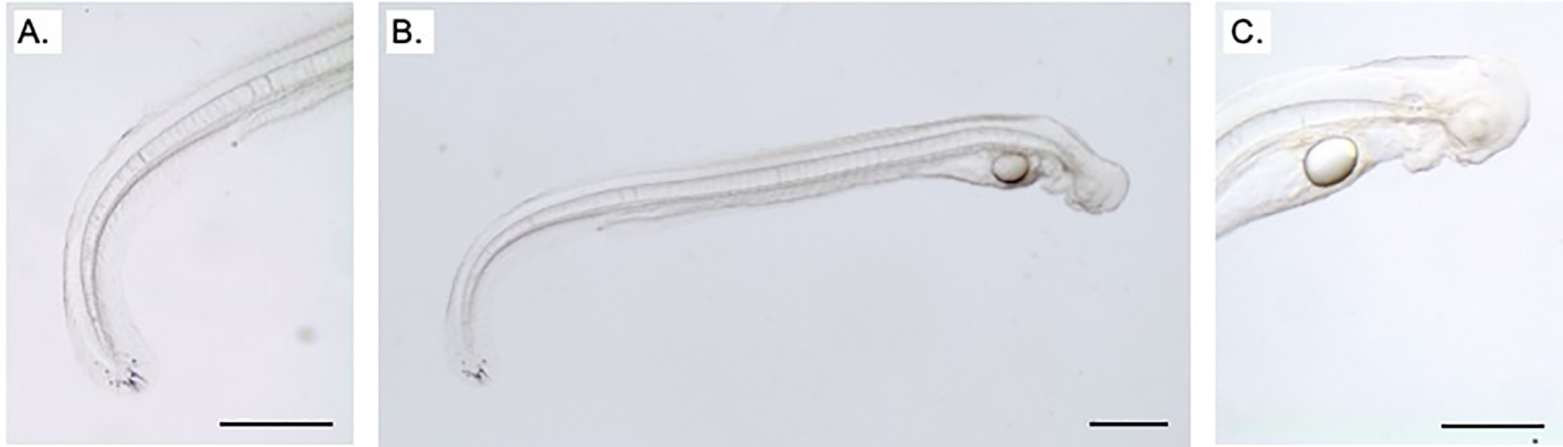
7 day old larvae. Micrographs of a larval *Anguilla australis* 7 days post hatch. Pigmentation is visible on the tip of the tail (A), the yolk sac is depleted (B), but the jaw/teeth did not develop (C). Scale bar = 500 um.

Larvae were reared in 10 cm petri dishes at ambient room temperature (range: 8–25 ^o^C). A number of different approaches to larval rearing were pitted against each other (i.e., the use of Leibovitz L15 incubation media, eel ringer and seawater; the use of a layer of agarose compared to a plastic petri dish bottom; daily *versus* twice weekly water changes) with no observable differences. Pigment was visible at the tip of the larva’s tail by 5 days post-hatch. Larvae were kept alive up to 7 days post-hatch by which time they were approximately 6 mm in length and their yolk sacs were depleted, but there were no signs of jaw/teeth formation (Fig 8).

## Discussion

Previous work incorporating androgens into traditional hypophysation methods fuelled our investigation into combined estrogen and androgen pre-treatment. In terms of ovarian development, androgens, in particular 11KT, have been associated with growth and lipid accumulation of previtellogenic oocytes [30–33]. Whilst androgens look to be the main driver reducing the time and amount of SPH required for induced maturation, estrogens could be instrumental for improving egg quality. Indeed, co-treatment of androgen and estrogen was previously seen to affect yolk accumulation in oocytes of shortfinned eels on the one hand, and to modulate plasma sex steroid levels to values deemed comparable to those in wild eels on the other [26].

Treatment of *A*. *australis* with high doses of androgens (30 mg of 17MT) resulted in significantly larger oocytes with abnormally high lipid accumulation [19]. No mention of oocyte abnormalities was conveyed following induced maturation of the European eel using a lower dose of 1 mg 17MT [20,34]. In *A*. *australis*, treatment with 1 mg 11KT did not result in any increase in lipid content within spawned eggs [19]. In the present study, all oocytes appeared morphologically similar and had comparable oocyte diameters at the pre-ovulatory stage regardless of steroid treatment. Together, these results indicate that the dose, rather than the type of androgen may most strongly affect oocyte cytology.

Studies on the Japanese eel (*Anguilla japonica*) using pharmacologically high doses (50–75 μg/g BW) of 17MT [35,36] reported oocytes in the advanced yolk vesicle stage. This research appears to be an anomaly as most eel researchers have reported that androgen treatment alone does not induce observable yolk sequestration in eel oocytes, regardless of the potential for some androgens to be aromatized and of the known role of E_2_ in stimulating hepatic vitellogenin production [21,22,24,25].

Steroid co-treatment resulted in an astonishing and as-of-yet unexplained interaction between 11KT and E_2_. As expected, treatment with either 11KT or E_2_ resulted in pharmacologically high levels of steroid circulating in the bloodstream (92 ± 4.8 ng/ml 11KT; 80 ± 8.7 ng/ml 0.2 mg E_2_; 105 ± 7.6 ng/ml 2 mg E_2_). However, co-treatment resulted in both steroids being present in physiologically appropriate concentrations (see: [26] for details). This perplexing outcome is currently under study but further reinforces the notion that combined treatment of E_2_ and 11KT could well result in a closer mimicking of maturation in captive eels to that in their naturally maturing counterparts.

In wild eel populations, vitellogenesis progresses relatively quickly compared to the many years required for pre-vitellogenic oocyte growth. Vitellogenesis occurs almost entirely during the oceanic spawning migration of freshwater eels as indicated by the pre-vitellogenic (*A*. *anguilla*: [37]; *A*. *japonica*: [35]) or early vitellogenic stage (*Anguilla rostrata*: [38]; *A*. *australis* and *Anguilla dieffenbachii*: [39]) of oocyte development in eels leaving the continental fresh water. Although the spawning grounds of many of the anguillid species have been identified [40–42], the exact duration of the migratory journey (and therefore the duration of vitellogenesis) is still being refined due to the difficulties tracking adult migration routes and identifying timing of spawning. However, based on when Japanese eels are known to start their migration, and the presence of either eggs and/or leptocephali in the water column, researchers estimate that the spawning migration, at least for that species, is completed in approximately six months [43–45]. The efficacy of androgens in compacting a six-month process to one less than a quarter in duration has the potential to be detrimental if yolk deposition proves inadequate. Despite these concerns, co-treatment of SPH with 17MT improved fertilization and hatch rates in *A*. *anguilla* [20]. It is possible, albeit not proven, that aromatization of 17MT into 17-methylestradiol-17β aided in the production of vitellogenin that became readily available for rapid uptake via receptor-mediated endocytosis. We previously demonstrated that combined pre-treatment of 11KT and E_2_ led to the formation of yolk granules in the oocytes before hypophysation commenced (see: [26]). We have not measured vitellogenin content in the eggs spawned from individuals in this treatment group or accurately assessed fertilization or hatch rates; however, our findings hold great promise for further improvements in egg quality and merit future investigations. It would be of considerable interest to compare pre-treatment with 11KT and E_2_ against pre-/co-treatment with 17MT in terms of traditional egg quality indicators (fertilization and hatch rates), fecundity and cost savings (both monetary and time).

Initial intentions to assess fertilization rates (as a proxy for egg quality) across the different treatment groups were thwarted due to the lack of motile sperm available when the first female eels spawned. Despite not being able to gather accurate fertilization rates of eggs spawned from any of the 11KT treated females, our results from the E_2_ treated females indicate an increase in fertilization rates with increased E_2_ dose. Of note, on several occasions, females treated with E_2_ (different doses) were spawning at the same time, i.e., the same pool of sperm could be used and fertilization rates for some females are therefore directly comparable. Timing is important when comparing fertilization rates as significant changes in milt volume and sperm density have been reported following hCG-induced testicular maturation [46]. Although research on the European eel indicates that 17MT treatment increased fertilization rates [20], no difference in the percentage of buoyant eggs obtained between control and 17MT-treated eels was reported. It is possible that because the 17MT-treated eels spawned significantly sooner than their control counterparts, there were substantial differences in the quality of sperm making true comparisons in fertilization rates difficult.

We identified a decrease in the HSI of spawned eels that had been pre-treated with E_2_. In contrast, Japanese eel treated simultaneously with E_2_ and SPH displayed an increase in HSI [47]. In the latter study, eels were not taken through until spawning, instead being dissected when eggs reached the tertiary yolk stage at a GSI of 10% after twelve weeks of injection. An initial increase in HSI is to be anticipated as lipids and proteins are packaged and mobilized to be incorporated into the developing oocytes. However, as oogenesis progresses and the energy stores become depleted, a decrease in HSI can be expected. Indeed, a decrease is seen in the natural cycles of many fish species at, or shortly after, spawning (e.g. Arctic cod (*Boreogadus saida*): [48]; pike (*Esox Lucius*): [49]; red gurnard (*Chelidonichthys kumu*): [50]; carp (*Cyprinus carpio*): [51]).

Estradiol pre-treated eels induced to mature may therefore be more closely resembling naturally maturing eels and arguably, may sequester more appropriate amounts of vitellogenin. Given the links between appropriate yolk formation and egg quality (reviewed in: [52]), and the low quality of eggs produced by standard hypophysation protocols, comparing vitellogenin content in the eggs stripped from E_2_ treated fish to that in eggs from SPH only treated fish seems worthwhile.

The frequency of attempts at induced spawning and larval rearing in shortfinned eels lag behind those in European and Japanese eels, with reliable protocols for larval rearing yet to be explored. Future hypophysation studies should refine larval rearing techniques in order to accurately determine the effects of steroid pre-treatment on larval survival. Regardless, the advances outlined in the current study, and summarised here, can be applied to freshwater eels in general and are unlikely specific to shortfinned eels. Whilst androgens appear to drive most of the desired outcomes (reduced fish handling, reduced time until spawning and reduced amounts of SPH), combined estrogen/androgen treatments likely pose significant advantages over androgen-only treatments in terms of yolk accumulation and therefore possible improvements in eggs quality. By combining the key bioactive metabolites of testosterone (E_2_ and 11KT), we were successful in inducing vitellogenesis in captive shortfinned eels in the absence of exogenous gonadotropins ensuring an advantageous starting point for hypophysation. Our findings reinforce the value for the ongoing search for refinement of induced spawning protocols in captive freshwater eels.

## Supporting information

S1 Video Hand-stripping of eel. Female eels (*Anguilla australis*) were hand-stripped and eggs collected prior to fertilization.
(MP4)
